# Investigating the Knowledge of Prebiotics, Probiotics, and Synbiotics That May Help to Improve the Gut-Organ Axis Function in Middle-Aged and Older Adults

**DOI:** 10.7759/cureus.66994

**Published:** 2024-08-16

**Authors:** Desiree Fermin, Sahar Alshammari, Joao Morgadinho, Tyler Halverson, Saifal Anwar, Ambikaipakan Senthilselvan, Kannayiram Alagiakrishnan

**Affiliations:** 1 Pharmacy, University of Alberta, Edmonton, CAN; 2 Medicine, University of Alberta, Edmonton, CAN; 3 Geriatrics, University of Alberta, Edmonton, CAN; 4 General Internal Medicine, University of Alberta, Edmonton, CAN; 5 Public Health, University of Alberta, Edmonton, CAN

**Keywords:** knowledge, gut biotics, synbiotics, prebiotics, probiotics

## Abstract

Background and aims: The use of gut biotics, including probiotics, prebiotics, and synbiotics, has shown substantial potential in the management of various health conditions possibly through the gut-organ axis. The role of gut biotics in modulating the gut-brain axis is becoming evident with more research focusing on this intervention. Improvement of gut-organ axis function is possible by using food-related products called gut biotics. However, there is limited comprehension of the knowledge and use of these intestinal or gut biotics. Our aim was to recognize knowledge gaps and assess the improvement of understanding following an education intervention.

Methods: A single-arm study encompassing a convenient sample of 161 inpatient and outpatient subjects aged 50 years and older was conducted at the University of Alberta Hospital from June to August 2023. Knowledge about gut biotics was evaluated using a structured questionnaire consisting of 16 questions and involving six thematic areas. To ensure validity, the questionnaire was pre-tested on 10 physicians and residents who were not part of the study. The questionnaire was administered to study subjects prior to receiving an information sheet about gut biotics. Two weeks after receiving the information sheet, all participants were contacted by phone, and the same questionnaire was administered again. Of the 161 patients, 122 completed the pre-intervention and post-intervention questionnaires and were considered in the analysis.

Results: The mean age of the participants was 72 years (SD: 10.8), 57% comprised women, and 39% had less than a high school education. The proportion of polypharmacy and multimorbidity was 87% and 97%, respectively. Following the intervention, there was a noticeable enhancement in knowledge across all the themes, with statistical significance (p<0.001) observed in 14 out of 16 questions as determined by the homogeneity statistical test.

Conclusions: Knowledge gaps in gut biotics were prevalent among study participants, and the educational intervention effectively contributed to the enhancement of knowledge. The results of this study provide valuable information for the development of targeted health education strategies focusing on gut biotics, which may play a role in improving gut-organ axis function.

## Introduction

The term "microbiome" refers to a microbial community (including prokaryotes, fungi, protozoa, and other microeukaryotes), microbial structures, metabolites/signal molecules, and mobile genetic elements, including transposons, phages, viruses, and relic DNA [1]. Dysbiosis is a condition where there is an imbalance of the gut microbiota, and this can lead to the development of many systemic diseases [2]. Imbalance may be seen as a reduction of good or beneficial bacteria, the growth of bad or harmful bacteria, or the loss of overall microbiome diversity. Gut biotics are food components that can affect gut microbes or live microbial administration or its products for therapeutic purposes [3]. There is emerging evidence that the gut microbiome (GM) and microbial dysbiosis contribute to various medical conditions. The intervention of the microbiota through intestinal biotics has shown a potential to manage various health conditions in different systems as well as mental health conditions by interactions with different organs through diverse pathways called the gut-organ axis including the gut-brain axis [4,5]. Correction of this microbial dysbiosis is possible with gut biotics. They include probiotics, prebiotics, and synbiotics. Probiotics are living microorganisms that produce health benefits. Prebiotics are nondigestible dietary components that reach the colon intact and promote the growth of beneficial bacteria. Synbiotics are a combination of prebiotics and probiotics that produce an overall synergistic effect. Overall, they are known as gut biotics. Dietary intervention and pharmacological supplements with gut biotics help correct the imbalance of the gut microbiota. This is done by repopulating with beneficial microorganisms that cause a decrease in intestinal permeability, inflammation, and alteration of metabolic activities. Despite this, there is limited understanding of the utilization of these gut biotics among middle-aged and older adults. The use of probiotics, prebiotics, and synbiotics, especially as food products or dietary supplements, is common. Current knowledge shows that it has a beneficial effect on a wide range of health conditions. Gut biotics are becoming popular dietary supplements after vitamins and minerals.

Current evidence from research studies showed that gut biotics have the potential to treat and prevent many medical conditions, including neurocognitive and some psychiatric disorders [4,5]. To our knowledge, no study has identified the knowledge gaps about gut biotics and their use in older subjects. With the emergence of research evidence showing the effectiveness of intestinal biotics in different medical conditions, these products could serve as attractive biotherapeutics (in addition to pharmacotherapeutics). New-generation probiotics (NGPs) research shows promise in colitis, inflammatory bowel disease (IBD), obesity/metabolic syndromes, diabetes mellitus, liver diseases, cardiovascular diseases, cancer, and neurodegenerative diseases. In addition, the research on the synergistic effects of these products with prebiotics, postbiotics, or traditional drugs is also being explored. Moreover, delivery of these compounds using nanoparticles and time-release formulations is underway to ensure targeted delivery to specific gut regions or cell types [6-8]. Many studies have addressed so far about different classes of gut biotics [9], and these studies have limitations due to sample size and also about treatment duration, but future studies should address these concerns.

For a change in healthy dietary behavior to occur [10], a good grasp of nutrition knowledge is very important. A study [11] showed that people with higher education can gain nutrition knowledge easily and correctly. The effect of gut biotics on human health has been clearly shown in the scientific literature, whereas the knowledge gaps have not been studied to a greater extent. The lack of knowledge about gut biotics, along with the growing evidence supporting their efficacy, highlights the need to educate the public and healthcare providers about it [12,13]. Knowledge surveys [14] could provide information on the needs, issues, and challenges of developing effective public health programs. Puspitasari et al. showed that knowledge has the potential to influence attitudes and good practice [15].

The objective of this study is to survey middle-aged and elderly subjects with respect to their knowledge, awareness, and understanding of the usefulness of probiotics, prebiotics, and synbiotics through the use of a questionnaire and also to do an intervention with an information sheet to improve the knowledge of these gut biotics. In recent years, with the growing knowledge about this type of biotherapeutics, in various medical conditions, a good understanding of this management is important. 

## Materials and methods

A single-arm study aimed at getting an understanding of the gut biotics knowledge was conducted. Single-group studies and those that evaluate a single intervention given to all subjects were included in the study design [16]. In this study, a single group of subjects was included in which all subjects received an intervention, and the outcomes were assessed pre- and post-intervention. As part of the intervention, subjects received an information sheet about gut biotics.

All the patients who were either inpatient or outpatient between May 2023 and August 2023 at the University of Alberta Hospital were considered for the study. Subjects with acute illness at the time of assessment, psychotic illness, and a history of moderate to severe dementia were excluded. Additionally, subjects with challenges in English communication and those who did not want to consent or were unable to consent were excluded from the study. After the exclusion, 161 patients who agreed to participate were included in this single-arm study. Physicians involved in the care of older adults assessed the decision-making capacity for inclusion and exclusion criteria. Initially, participants completed a questionnaire on demographic characteristics and a structured questionnaire on gut biotics. This was followed by an educational intervention in the form of an information sheet. The information sheet was prepared by the physicians and the dietician in the study. With an information sheet or leaflet, a patient can understand the information about gut biotics much more clearly. A study by Adepu and Swamy showed that knowledge gain is very important to create a greater impact on disease management. With this, patients are empowered to make independent decisions and have a level of responsibility in their care. Patient information sheets with additional information can create this impact [17]. Information sheets are globally accepted patient counseling aids, which can help to improve the health literacy of the individual [18]. After a two-week period, all participants were contacted by phone, and the same questionnaire on gut biotics was administered. Information on the number of medications and comorbidities was obtained from the medical record.

The structured questionnaire on gut biotics (Appendix 1) was originally prepared by the physicians and the dietician in the study and was composed of 16 questions separated into six thematic areas which included familiarity or awareness, understanding the terminology, knowledge of gut biotics and their health benefits, current consumption and future use, biotics and food products, and lifestyle and gut biotics. All questions were closed-ended from where both quantitative and qualitative information were collected. Responses included dichotomous and Likert scale responses. To ensure validity, the questionnaire was pre-tested in 10 individuals which included physicians and residents who were not part of the study. All coinvestigators, including a dietician, participated in the preparation of the information sheet (Appendix 2) for the intervention and questionnaire (Appendix 1). Polypharmacy was defined as the intake of five or more medications. Multimorbidity was defined by the presence of two or more comorbidities. Ethics approval was obtained from the University of Alberta Health Research Ethics Board (approval number: Pro00129160).

Statistical analysis

Frequencies and percentages were used to describe demographic characteristics, polypharmacy, and comorbidities. The pre- and post-responses to the question in the structured questionnaire were initially compared using an asymptotic symmetry test. An average score was obtained for each thematic area after coding the correct responses to 1 and the incorrect responses and the "Don't know" responses to 0. The mean and standard deviation of the score for each thematic area were determined, and the paired t-test was used to test the differences between the post- and pre-intervention scores of the thematic areas. The "Don't know" responses on the Likert scale were coded as 0. Multiple regression analysis was used to examine the association between the differences in the post- and pre-intervention thematic scores; education, multimorbidity, and polypharmacy after controlling for pre-intervention thematic scores; and age group and gender. Statistical analysis was conducted using Stata Statistical Software: Release 18 (2023; StataCorp LLC, College Station, Texas, United States), and statistical significance was set at p<0.05.

## Results

Of the 161 patients, 122 completed the pre-intervention and post-intervention questionnaires, with 24 dropping out and 15 being deceased. As shown in Table 1, the mean age of the study participants was 72.2 years (SD 10.8), 70 (57.4%) were female, and 48 (39.3%) had less than a high school education. Polypharmacy and multimorbidity were present in 86.9% (n=106) and 96.7% (n=118) of the participants, respectively. The most common comorbidity among participants was cardiovascular disease (n=94, 77.1%) followed by gastrointestinal disease (n=78, 63.9%) and musculoskeletal disease (n=67, 54.9%).

**Table 1:** Characteristics of study participants and prevalence of comorbidity and polypharmacy

	(n)	(%)
Age group		
50-64 years	36	29.5
65-80 years	52	42.6
81 years and above	34	27.9
Gender		
Male	52	42.6
Female	70	57.4
Education		
Unknown	13	10.7
Less than high school	48	39.3
High school	5	4.1
Some post-secondary	13	10.7
Post-secondary	43	35.3
Polypharmacy		
No. of medications ≤5	16	13.1
No. of medications >5	106	86.9
Dyslipidemia		
Absent	75	61.5
Present	47	38.5
Cardiovascular condition		
Absent	28	23
Present	94	77.1
Respiratory condition		
Absent	71	58.2
Present	51	41.8
Gastrointestinal condition		
Absent	44	36.1
Present	78	63.9
Neurological condition		
Absent	56	45.9
Present	66	54.1
Musculoskeletal condition		
Absent	55	45.1
Present	67	54.9
Genitourinary condition		
Absent	70	57.4
Present	52	42.6
Cancer diagnosis		
Absent	90	73.8
Present	32	26.2
Multimorbidity		
Comorbidity <2	4	3.3
Comorbidity ≥2	118	96.7

The significant differences in the proportion of responses for the 16 questions in the questionnaire between pre- and post-intervention were tested using the asymptotic symmetric tests which allowed for correlated responses from the same patients. Following the educational intervention, there was a noticeable enhancement in knowledge across all the themes, with statistical significance (p<0.05) detected in 14 out of 16 questions (Table 2). A knowledge-based average score was determined for each thematic area, and the significant differences in the knowledge-based average score were tested using paired t-tests which allowed for the correlated responses from the same patients (Table 3). There were statistically significant changes (p<0.05) in knowledge-based average scores in all thematic areas except for the current consumption of fermented foods after the intervention (Table 3).

**Table 2:** Distribution of pre- and post-intervention responses to the questionnaire. P<0.05 is considered as statistically significant

Question	Pre-intervention		Post-intervention		P-value
	(n)	(%)	(n)	(%)	
Our body has both good and bad gut bacteria					
True	114	93.44	117	95.9	0.06
False	0	0	2	1.64	
Don't know	8	6.56	3	2.46	
How familiar are you with probiotics, prebiotics, and synbiotics?					
No knowledge	53	43.44	15	12.3	<0.001
Somewhat	51	41.8	54	44.26	
Average	12	9.84	47	38.52	
Quite	6	4.92	6	4.92	
Probiotic foods contain live bacteria					
True	49	40.16	105	86.07	<0.001
False	5	4.1	6	4.92	
Don't know	68	55.74	11	9.02	
Prebiotics provide the fuel that feeds gut bacteria					
True	40	32.79	97	79.51	<0.001
False	3	2.46	4	3.28	
Don't know	79	64.75	21	17.21	
Synbiotic products contain both probiotic and prebiotic contents					
True	12	9.84	86	70.49	<0.001
False	2	1.64	2	1.64	
Don't know	108	88.52	34	27.87	
Most healthy adults can safely add foods/food products that contain prebiotics, probiotics, or both to their diets					
True	76	62.3	112	91.8	<0.001
False	6	4.92	1	0.82	
Don't know	40	32.79	9	7.38	
Probiotic effects are seen in all fermented foods					
True	38	31.15	51	42.15	<0.01
False	26	21.31	41	33.88	
Don't know	58	47.54	29	23.97	
Gut health can have an impact on mental and physical health including chronic health conditions					
Disagree	1	0.82	1	0.82	0.03
Somewhat disagree	0	0	1	0.82	
Somewhat agree	13	10.66	5	4.1	
Agree	96	78.96	106	89.34	
Other	12	9.84	6	4.92	
Probiotics, prebiotics, and/or synbiotics have a possible role in treating mental health disorders and chronic medical conditions					
Disagree	2	1.64	1	0.82	<0.001
Somewhat disagree	4	3.28	0	0	
Somewhat agree	20	16.39	28	22.95	
Agree	43	35.25	79	64.75	
Other	53	43.44	14	11.48	
There is a connection between the brain and the gut					
True	100	81.97	119	97.54	<0.001
False	5	4.1	0	0	
Don't know	17	13.93	3	2.46	
Would you consider regularly consuming probiotics, prebiotics, or synbiotics (or one of them) as food products for a specific health benefit?					
Yes	83	68.03	118	96.72	<0.001
No	3	2.46	0	0	
Don't know	36	29.51	4	3.28	
Fermented foods can include probiotic bacteria such as yogurt, kefir, sauerkraut, miso, and tempeh					
True	71	58.2	113	92.62	<0.001
False	2	1.64	1	0.82	
Don't know	49	40.16	8	6.56	
Prebiotic foods include bananas, onions, asparagus, artichokes, and oats					
True	28	22.95	98	80.33	<0.001
False	5	4.1	1	0.82	
Don't know	89	72.95	23	18.85	
How often do you consume fermented foods currently?					
None	35	28.69	24	19.67	0.19
1-2 days/week	27	22.13	25	20.49	
3-4 days/week	12	9.84	22	18.03	
5-6 days/week	10	8.2	8	6.56	
Daily/multiple times	38	31.15	43	35.25	
Weekly					
How a fermented food is made, stored, and handled impacts the potential probiotic health benefit					
True	81	66.39	107	87.7	<0.001
False	3	2.46	2	1.64	
Don't know	38	31.15	13	10.66	
Antibiotics, alcohol, and/or smoking can damage or destroy good gut bacteria					
True	98	80.33	116	95.08	<0.01
False	3	2.46	1	0.82	
Don't know	21	17.21	5	4.1	

**Table 3:** Distribution of pre- and post-intervention thematic area scores. P<0.05 was considered as statistically significant

Questions by themes	Pre-intervention score	Post-intervention score	P-value
	Mean (SD)	Mean (SD)	
Familiarity or awareness			
2. How familiar are you with probiotics, prebiotics, and synbiotics?	0.76 (0.82)	1.36 (0.76)	<0.001
Understanding the terminology			
3. Probiotic foods contain live bacteria	0.82 (0.94)	2.36 (0.97)	<0.001
4. Prebiotics provide the fuel that feeds gut bacteria			
5. Synbiotic products contain both probiotic and prebiotic contents			
Knowledge about these products and their health benefits			
6. Most healthy adults can safely add foods/food products that contain prebiotics, probiotics, or both to their diets	2.25 (1.08)	3.07 (0.83)	<0.001
7. Probiotic effects are seen in all fermented foods			
8. Gut health can have an impact on mental and physical health including chronic health conditions			
9. Probiotics, prebiotics, and/or synbiotics have a possible role in treating mental health disorders and chronic medical conditions			
10. There is a connection between the brain and the gut			
Future and current consumption			
11. Would you consider regularly consuming probiotics, prebiotics, or synbiotics (or one of them) as food products for a specific health benefit?	0.68 (0.47)	0.97 (0.18)	<0.01
14. How often do you consume fermented foods currently?	1.90 (1.65)	2.17 (1.57)	0.20
Biotics and food products			
12. Fermented foods can include probiotic bacteria such as yogurt, kefir, sauerkraut, miso, and tempeh	0.81 (0.73)	1.73 (0.55)	<0.001
13. Prebiotic foods include bananas, onion, asparagus, artichokes, and oats			
Lifestyle and gut biotics			
16. Antibiotics, alcohol, and/or smoking can damage or destroy good gut bacteria	0.80 (0.40)	0.95 (0.22)	<0.001

In the multiple regression analysis, the association between the differences in the score for each question between pre- and post-intervention and the factors described in Table 1 was examined. There was a significant association (p<0.05) between the change in the post- and pre-intervention score for the question "synbiotic products contain both probiotic and prebiotic content" and education attainment. However, no significant association was observed between the differences in the post- and pre-intervention thematic scores and multimorbidity and polypharmacy. The average or more knowledge about probiotics, prebiotics, and synbiotics increased from pre-intervention (n=18, 14.8%) to post-intervention (n=53, 43.4%). Among those with average or more knowledge of probiotics, prebiotics, and synbiotics, the current consumption of fermented foods at least 1-2 days per week or more increased from pre-intervention (n=14, 77.8%) to post-intervention (n=46, 86.8%), whereas among those with average or more knowledge of probiotics, prebiotics, and synbiotics, the future regular consumption of probiotics, prebiotics, and synbiotics increased from pre-intervention (n=16, 88.9%) to post-intervention (n=52, 98.1%). The multimorbidity was prevalent among 118 (96.7%) of the participants. Among those with multimorbidity, the current consumption of fermented foods at least 1-2 days per week or more was 72% (n=85) during pre-intervention and 80.5% (n=95) during post-intervention, whereas the future regular consumption of probiotics, prebiotics, and synbiotics was 69.5% (n=82) during pre-intervention and 96.6% (n=114) during post-intervention with the difference being statistically significant.

## Discussion

The present study was conducted to examine the understanding of gut biotics and their use in middle-aged and older individuals. The study found that a considerable percentage (44%) of the subjects had no knowledge about gut biotics, which is quite significant considering recent advances in this field. These results underscore the need to educate the public about this topic. In the six thematic areas of the questionnaire, the most recognized themes are familiarity and awareness, understanding the terminology, knowledge about gut biotics and their health benefits, consumption and future use, biotics as food products, and finally the interplay between lifestyle and gut biotics.

This intervention can now be implemented in clinical practice for a variety of health conditions as more evidence continues to emerge. At this stage, it is highly important to focus on increasing public awareness. Through the application of knowledge translation in our study, using a handout about gut biotics, we discovered a significant improvement in knowledge. Unlike previous studies that mainly focused on probiotics, this is the first prospective study evaluating knowledge of gut biotics (probiotics, prebiotics, synbiotics).

Unlike most studies that focused on different medical professionals, this study included middle-aged and older adult patients as participants. Furthermore, this study is the first to demonstrate knowledge translation in older adults on this topic, improving their comprehension. In line with this, a recent article from the microbiome-related food systems working group emphasized the educational needs in this area [19]. Different studies have focused on the education of different healthcare professionals [20-23]. Until now, the only way the public can know information about this topic is through science or medical communication through journals [24].

The health benefits of the microbiota in humans can occur through dietary changes [25]. Some studies have been carried out showing the beneficial effects of eating foods rich in the human microbiota [26]. There is a need to improve public knowledge and increase awareness about microbiome and microbial therapeutics [27]. The field of microbial therapeutics is becoming more relevant with dietary supplements such as gut biotics, fermented foods, and starter cultures [25]. In this study, we also saw the desire to use intestinal biotics as food after a good understanding of intestinal biotics.

To assist patients who may be interested in using intestinal biotics in the future, healthcare professionals should provide informed and objective advice on the topic [28]. This study may help identify areas of focus for health education related to gut biotics, particularly food products. We have released a patient information sheet (Appendix 2) which primary care physicians can use in their offices to distribute to their patients who are interested in knowing information about gut biotics.

This is the first prospective single-group study with a pre- and post-test design to show the translation of knowledge on gut biotics in older adults. The limitations of this study are the use of a convenient sample size and the absence of control group subjects in this study. The disadvantages of this study include limited generalizability. In this study, we used middle-aged and older individuals as they are more prone to these chronic diseases, where gut biotics can have maximum benefit.

## Conclusions

Knowledge gaps in intestinal biotics were prevalent among study participants, and the educational intervention effectively contributed to the enhancement of knowledge. The results of this study provided valuable information for the development of targeted health education strategies focusing on gut biotics. This research underscored the importance of spreading knowledge in this domain to empower middle-aged and older adults with informed choices regarding gut biotics. This study may help identify areas of focus for health education related to gut biotics, particularly food products, especially useful for patients and healthcare professionals. Education and awareness to improve public knowledge about gut dysbiosis and gut biotics has the potential to improve the management of many medical conditions.

## Appendices

Appendix 1

**Table 4:** Survey questionnaire

Questionnaire
1. Our body has both good and bad gut bacteria
True
False
Don't know
2. How familiar are you with probiotics, prebiotics, and synbiotics?
No knowledge
Somewhat familiar
Average knowledge
Quite knowledgeable
3. Probiotic foods contain live bacteria
True
False
Don't know
4. Prebiotics provide the fuel that feeds gut bacteria
True
False
Don't know
5. Synbiotic products contain both probiotic and prebiotic contents
True
False
Don't know
6. Most healthy adults can safely add foods/food products that contain prebiotics, probiotics, or both to their diets
True
False
Don't know
7. Probiotic effects are seen in all fermented foods
True
False
Don't know
For the next two questions, please indicate how much you agree with the statements:
8. Gut health can have an impact on mental and physical health including chronic health conditions
Disagree
Somewhat disagree
Somewhat agree
Agree
9. Probiotics, prebiotics, and/or synbiotics have a possible role in treating mental health disorders and chronic medical conditions
Disagree
Somewhat disagree
Somewhat agree
Agree
10. There is a connection between the brain and the gut
True
False
Don't know
11. Would you consider regularly consuming probiotics, prebiotics, or synbiotics (or one of them) as food products for a specific health benefit?
Yes
No
Don't know
12. Fermented foods can include probiotic bacteria such as yogurt, kefir, sauerkraut, miso, and tempeh
True
False
Don't know
13. Prebiotic foods include bananas, onion, asparagus, artichokes, and oats
True
False
Don't know
14. How often do you consume fermented foods currently?
None
1-2 days per week
3-4 days per week
5-6 days per week
Daily or multiple times per day
15. How a fermented food is made, stored, and handled impacts the potential probiotic health benefit
True
False
Don't know
16. Antibiotics, alcohol, and/or smoking can damage or destroy good gut bacteria
True
False
Don't know

Appendix 2

Patient Information Sheet

The gut or food passages have many types of bacteria. Bacteria are thought of as being bad for your health and causing illness. Both good and bad bacteria exist inside your gut. More balanced gut bacteria can improve and protect your health. The wrong balance of good and bad bacteria may cause many health disorders, such as diabetes and heart diseases. Bacteria can communicate with other body parts, like the brain, through the "gut-brain axis" and influence your mental health. Diet, some medications, and lifestyle can affect gut bacterial health (good and bad). Alcohol, smoking, and some medications can harm gut bacterial health. Gut biotics can help you get a good bacterial balance through food. Gut biotics can improve health through probiotics, prebiotics, or synbiotics, which will be described below. Probiotics are live forms of bacteria that give you health benefits when you take an adequate amount. Eating fermented foods is a way for our body to get probiotics. These foods have been around for thousands of years and are consumed by many people around the world. Examples of fermented foods include yogurt, kombucha (tea), kefir (a milk drink), some pickles, sauerkraut, kimchi, miso, tempeh (soybean food), and others. Live bacteria in food products can be harmed by cooking, poor storage, filtering, and direct sunlight. This can change the health benefits of these foods.

Probiotic foods are safe for most adults to eat. With probiotics, problems may arise in persons with severe sickness. There have been a few case reports of superinfections in such persons. Probiotics are frequently used with few incidents of such infections. This backs up the general safety of these foods when taken in reasonable amounts. Prebiotics are a fuel source for the gut bacteria that is good for your health. This fuel can include fiber, sugars your body can't break down. Examples of prebiotics include bananas, onions, asparagus, artichokes, oats, and others. Prebiotics are thought to be safe for most people. Some people may have problems digesting certain prebiotic foods which can cause some gas, bloating, or discomfort. Every person will find foods they enjoy and foods that they wish to avoid. Synbiotics are products that are a mix of both a prebiotic and a probiotic. Both can then work alone or together with the goal of improving good gut bacteria and health overall.

If you have a weak immune system or are sensitive to gut biotics, talk to your healthcare expert first. Medications like antibiotics can sometimes affect gut health in ways you may not want. It is key to take needed medicines as guided by the medical team. If you want to change your medications, talk to your healthcare providers first. Research points out that gut biotics may help to treat and prevent many medical conditions including cognitive and mental disorders.

If you need more information about this which can play a role in your overall health and well-being, please contact your healthcare provider (primary care physician or dietician) who will be able to provide more information and guide you in the right direction.
